# Mineralized collagen artificial bone repair material products used for fusing the podarthral joints with internal fixation—a case report

**DOI:** 10.1093/rb/rbx015

**Published:** 2017-06-23

**Authors:** Nihar S. Ghate, Helen Cui

**Affiliations:** 1 Division of Foot and Ankle Surgery, Indiana Regional Medical Center, Indiana, PA 15701, USA; 2 Beijing Allgens Medical Science and Technology Co., Ltd., Beijing 100176, China

**Keywords:** medial column, metatarsophalangeal joint, navicular-cuneiform fusion, mineralized collagen

## Abstract

In this study, we reported a case with collapse and subluxation of metatarsal-cuneiform joint, navicular-cuneiform joint with subluxed the right first metatarsophalangeal joint. The injured medial column was internally fixed with compression arthrodesis. The fusion site was firmed up with BonGold® Bone Sponge and Bone Putty. The prognosis of fused navicular-cuneiform joint and metatarsal-cuneiform joint were examined by X-ray shortly after surgical operation and followed up 2, 4, 6, 9 and 13 weeks after the surgical operation. The medial column was perfectly fused by compression arthrodesis. These results justified and favored the application of mineralized collagen as an excellent alternative to autograft in fusing the podarthral joints with internal fixation.

## Introduction

In cases of severe collapse and subluxation of podarthral joints, fusing the affected joints is often an inevitable approach to provide mechanical buttress and relieve the pain. The orthopedic surgeons have adopted varieties of natural and artificial materials supplementary to internal fixation and compression arthrodesis and been evaluating their advantages and shortages through clinical practice. The mineralized collagen is a novel bone substitute developed by biomimetic strategy that mimics the extracellular matrix of natural bone both in chemical composition and in microstructure. It can avoid donor site morbidity and complications caused by harvesting autologous bone graft. Thanks to its highly bionic microstructure and bioactivity qualities, mineralized collagen induces ideal osteogenesis and intramembranous ossification. Here we reported the first application of mineralized collagen in podarthral joint defect repair.

## Case report

**Figure 1:**
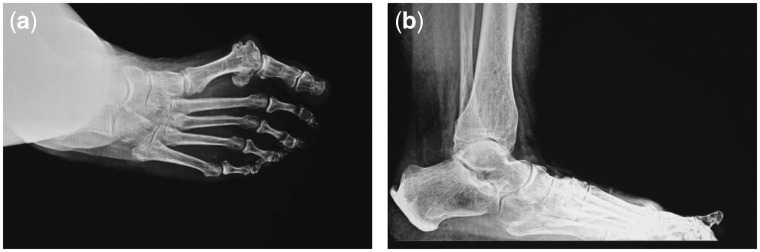
Transverse and sagittal section preoperative X-ray examination. Subluxated and collapsed medial arch was found out at the navicular-cuneiform, metatarsal-cuneiform joint with subluxed first MTPJ

A 59 years old female visited the clinic with ongoing pain located to the right first metatarsophalangeal joint (MTPJ) and medial column. She stated the pain had been ongoing for 2–3 years and has gradually gotten worse recently. Through visual analogue scale, she rated the pain as 7/10. She recently had problems with arthritic changes in her body and felt the same pain in her feet. She stated that the pain in her feet was not significantly bad until recently. The patient has past medical history of thyroid disorder and family history of diabetes and heart disease. Pertinent physical exam revealed some problem in musculoskeletal system. The right lower extremity has pain on palpation to the first MTPJ with a large dorsal prominence as well as pain at the first metatarsal-cuneiform joint with hypermobility. Pain was also elicited throughout the navicular-cuneiform joint. Subluxation and collapse in the medial arch were found out at the navicular-cuneiform joint with subluxed first MTPJ (Fig. 1). Pain was tracked within the first MTPJ with a range of motion in dorsiflexion and plantar flexion of first MTPJ. The left lower extremity had deviated first MTPJ with minimal dorsal medial eminence. No pain was elicited either upon palpation or in range of motion of the joint. Patient ambulated well, however, with significant pain throughout the right lower extremity, especially the medial column of gambrel. Another diagnosis was bilateral hallux valgus arthrosis.

Although the patient understood she had a significant deformity, surgeons informed that they could try conservative measures first if the patient was not ready for reconstructive surgery. The patient stated that she would rather have the surgery now while she was healthy rather than waiting and being too enervated to undergo the surgery. The surgical plan was hence sketched to include a Lapidus procedure with compression fusion of metatarsal-cuneiform joint and navicular-cuneiform joint. To heal the operative wound, patient should be non-weight bearing post operatively for 6–8 weeks.

**Figure 2:**
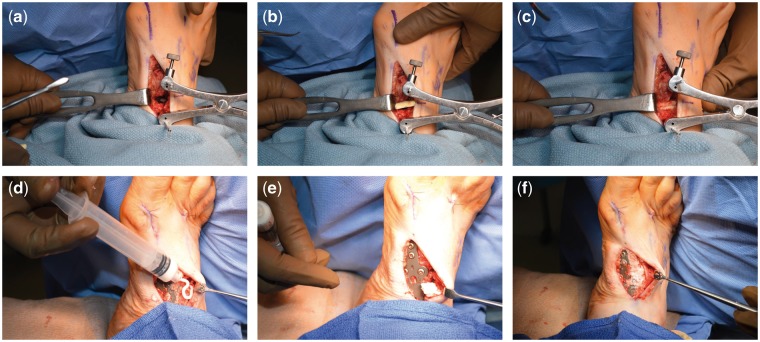
Surgeons applied the BonGold^®^ Bone Sponge and BonGold^®^ Bone Putty to the injured navicular-cuneiform joint. From a to c, surgeons opened navicular-cuneiform joint with a distracter and then inserted the BonGold^®^ Bone Sponge into the joints piece by piece. From d to f, the injured medial column was permanently stabilized. After internal fixation with plate and screws, the fusion site was firmed up with BonGold^®^ Bone Putty

**Figure 3:**
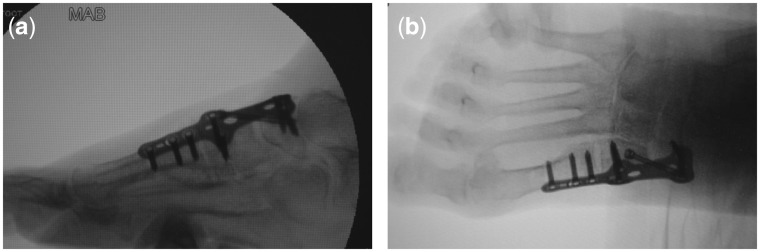
Transverse and sagittal section X-ray examination after surgical operation. Metatarsal-cuneiform joint and navicular-cuneiform joint of right lower extremity was perfectly stabilized with an internal fixation plate and screws shortly after surgical operation

**Figure 4:**
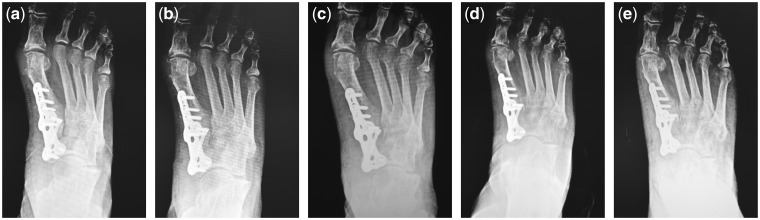
Follow-ups of clinical outcomes by X-ray examinations. Transverse section X-ray examinations at the 2nd, 4th, 6th, 9th and 13th weeks after surgical operation were displayed from a to e, respectively. The metatarsal-cuneiform joint and navicular-cuneiform joint of right lower extremity was perfectly stabilized with an internal fixation plate and screws. On part e, we can see complete consolidation of the medial column with excellent coalescence at the medial column without any lucency visible at the fusion sites. This is one-and-half times faster than expected for this type of surgery

Brief operative synopsis was as follows. The surgeons employed mineralized collagen artificial bone repair material products named Bongold^®^ patented by Beijing Allgens Medical Science and Technology Company [1]. Bongold^®^ is superior to kindred products in its high performance to induce bone regeneration and osteogenesis [2, 3]. Having high performance in repairing bone defects in orthopedics, Bongold^®^ won the FDA market access license (K141725) in July 2015. With mineralized collagen of BonGold^®^ Bone Sponge and BonGold^®^ Bone Putty, the medial column in the right lower extremity was surgically fused with internally fixation. We began with incision over the metatarsal-cuneiform joint and the navicular-cuneiform joint. Once down through the periosteum the joints were immobilized. Hereafter, the surgeons opened both joints with a Hintermann Style Distractor and then propped these joints. Next, the surgeons saturated BonGold^®^ Bone Sponge with normal saline and inserted them into both of these joints piece by piece (Fig. 2a–c). Then the surgeons released the distractor to allow for proper fitment. The injured medial column was temporary stabilized and then permanently stabilized with an internal fixation plate and screws. Finally, the fusion site firmed up with BonGold^®^ Bone Putty (Fig. 2d–f). The outcome of arthrodesis in metatarsal-cuneiform joint and navicular-cuneiform joint was examined by X-ray shortly after surgical operation (Fig. 3). The curative effect was followed up at the point of 2 weeks, 4 weeks, 6 weeks, 9 weeks and 13 weeks after the surgical treatment (Fig. 4). The medial column was perfectly fused by compression arthrodesis.

## Discussion

Despite the ground rapidly gained in joint surgery, most of these once prevalent synthetic bone substitutes such as bioceramic ones are not widely applicable any more due to inherent shortcomings. In most cases of arthrodesis, the joints need to coalesce into fused bones and permanently fix the subluxation or abarticulation. Internal fixation and screws are extensively practicing in compression arthrodesis [4, 5]. Without suitable biomaterials, surgeons have to fall back on osteotomy or autograft and inevitably bring about severe complications such as deformity and dysfunction in the donor site [6, 7]. The failure rate is not high, but some cases may require additional surgery, and the outcome of podiatric rehabilitation may be affected. With special attention to risk factors and their connection to the principles of prevention and treatment, surgeons care more and more about the major long-term complications, reliable and safe implant, rhythm of resorbability and measures implemented in practice to minimize the sequelae [8, 9]. As a result of these concerns, biomaterials of choice entail attributes of excellent osteoconductivity, biocompatibility and additional properties like radiolucency, antibiosis, free of immunologic rejection, poor thermal conductivity and ability to induce osteanagenesis [10]. Fracture, non-union, collapse, subluxation and any other joint injury requiring reparative regeneration of osseous tissues indicate application of mineralized collagen artificial bone repair material products that are endued with all the merit of an ideal biomaterial.

With crystallized hydroxyapatite arrayed along fibrils of type I collagen, mineralized collagen fabricates the understructure of the hardest human connective tissues such as bone and dentin [10, 11]. Within the mineralized collagen, hydroxyapatite crystals with size on nanometer scale orderly juxtapose along fibrils fabricated from type I collagen in a specific hierarchically staggered nanostructure. The mineral components in the form of nanometric crystals account for two-thirds of the dry weight in the bone matrices and one-third is formed by collagen fibrils [12]. The inorganic phase in the composite is carbonate substituted by hydroxyapatite with a low crystallinity. Mineralized collagen provides the calcified tissue with essential sterical niche for the maintenance, proliferation and ossification effect of osteocytes. Besides microstructures, all the trivial physiological functions of compact bones and trabeculae derive from mineralized collagen.

The rationale for the assembling process of mineralized collagen has been explained by self-assembly process [13]. Guided by biothermodynamics and related principles, synthetic biomaterials mimicking natural structure of mineralized collagen have been flourishing [14]. The mineral crystals with size on nanometer scale uniformly precipitate and disseminate along the fibril matrix. The composite is nearly identical to the nanostructure of osseous tissues in both chemical composition and microstructure to the largest extent.

Other than traditional synthetic bone grafts that only act as structural supersedes without any functional bioactivity, the mineralized collagen is not only biocompatible, biodegradable and absorbable but also osteoconductive and osteoinductive. Prostheses made of mineralized could undergo ‘creeping substitution’, a process of contact osteogenesis and bone regeneration after implantation [14]. A number of *in vitro* and *in vivo* studies have proven the bioactive and biodegradable characteristics of this bone-resembling material [15]. In certain case, favorable treatment effects were achieved by simple implantation of the mineralized collagen bone graft. In the implantation process, the minimal invasion can be achieved to protect the soft tissue and blood circulation [16]. Other case demonstrated promising result regarding the efficacy of mineralized collagen as an extender in displaced intra-articular calcaneal fractures with successful healing rate and clinical scores equivalent to those of autograft. Mineralized collagen may be a good alternative to autograft in displaced intra-articular calcaneal fractures with trabecular defects [3].

In conclusion, mineralized collagen materials were applied to the gambrel arthrodesis by being implanted into the collapsed and subluxated metatarsal-cuneiform joint and navicular-cuneiform joint. To evaluate the clinical outcomes, the case was followed up for 13 weeks. The joints fused very well with the mineralized collagen without inflammatory responses, itching or exudation at the surgical sites. The clinical outcomes indicate that mineralized collagen is effective for the podarthral joint fusion surgical operation.
